# A Strategy for Nonmigrating Highly Plasticized PVC

**DOI:** 10.1038/s41598-017-10159-7

**Published:** 2017-08-24

**Authors:** Jun Yuan, Bin Cheng

**Affiliations:** Key Laboratory of Beijing City on Preparation and Processing of Novel Polymer Materials, Beijing University of Chemical Technology, Beijing, 100029 China

## Abstract

Nonmigrating highly plasticized PVC was prepared based on a new compound that acts as a plasticizer that was derived from di(2-ethylhexyl) 4-hydrophthalate and chlorinated paraffin-52. The as-prepared PVC has a plasticizing efficiency as high as DOP and its migration is totally suppressed. Unlike other reported methods, this approach increases the interaction between phthalate and PVC to suppress its migration, not simply to enlarge its molecular size (or molecular weight). This methodology is highly versatile for producing the desired non-leaching PVC with a permanent plasticizer effect.

## Introduction

Poly(vinyl chloride) (PVC), as the second largest polymeric material on the market^1^, is widely used in all areas of human activity, for example, building materials, medical devices, food packaging, clothing and toys. However, PVC is a strongly polar polymer in which force between molecules is very strong. To show plasticity, PVC must be heated to a certain temperature, which is challenging for PVC moulding products. Therefore, in PVC materials, plasticization is necessary to reduce softening temperature, decrease melt viscosity and increase mobility to improve its processing properties and product flexibility^2^. Plasticization can be achieved internally by introducing into the original polymer a comonomer, which reduces crystallizability and increases chain flexibility, or externally by compounding PVC with a low molecular weight compound. The low molecular weight compound was defined as a plasticizer by The Council of the International Union of Pure and Applied Chemistry. Internally plasticized PVC can maintain its performance over long-term use because there is no plasticizer migration. However, internal plasticization is less efficient and generally has an unsatisfactorily narrow use temperature range. Using external plasticizers, it is very convenient to select from a variety of plasticizers depending on the desired properties and processability of PVC. Plasticizer is not only a processing aid but also an important component to determine the performance and application of PVC. Therefore, plasticizers are the most important commercial application of PVC. However, plasticizers can leach out of flexible PVC, changing the performance of PVC with age and contaminating the environment. Phthalate esters are still the most powerful plasticizers and dominate the plasticizer market due to their great plasticizing effect and low-cost, although phthalate plasticizers can migrate to the surface, leading to material performance degradation and a negative influence on human health^3–16^. In response to consumer and regulatory pressures, developing nonmigrating or phthalate-free PVC is very active in past few years. Recently, some other plasticizers instead of phthalate plasticizers, such as biocompatible material and oligomer^17–27^, have been reported without toxicity. However, compared to phthalate plasticizers, majority of them have a poor plasticizing effect and the plasticizer migration still cannot be avoided. Therefore, phthalates might be still used, but its leaking or migration should be prevented. A coating on the surface of PVC was reported to isolate plasticizer from the environment and prevent the leaking of phthalate from PVC plastics^28–31^. However, the surface coating costs are too high to be commercialized. Another potential approach is covalently attaching phthalate molecules to the main chain of polymers to suppress its migration. For example, Rebecca Braslau *et al*.^32^ attached phthalate molecules to all-carbon polymer backbone to develop new polymeric phthalates via (co)polymerization of 4-vinyl phthalate ester. Unfortunately, they did not try them to PVC. In general, their large molecule size hinders diffusion in PVC to suppress migration. However, the rate of diffusion of the plasticizer is one of the most important factors determining plasticizer efficiency^33, 34^. Polymeric plasticizer generally has low plasticizing efficiency. Navarro *et al*.^35^ replaced the chlorine on the PVC backbone via nucleophilic substitution of thiol groups attached to the benzene ring of di(2-ethylhexyl)phthalate (also known as dioctyl phthalate, DOP, the most common plasticizer), to give totally nonleachable PVC. Unfortunately, although the modified PVC came from a common plasticizer DOP, it had very poor plasticization performance and its glass transition temperature cannot fall below 20 °C without a ratio of attached DOP to PVC below 1.7:1. This is because DOP molecules have been fixed on the PVC backbone and lack the freedom to solvate and desolvate various attachments on the PVC molecule. While a low rate of diffusion provides high plasticizer permanence, it results in lower plasticizer efficiency, and vice versa^36^. No better methods involving migration/plasticizing efficiency balance for plasticizers have been documented.

In this study, we attached DOP to chlorinated paraffin (CP), acting as a secondary plasticizer in PVC, to prepare a new compound giving PVC highly plasticization without migration. Unlike other reported methods, our approach covalently attaches phthalate to chlorinated paraffin, a low molecular weight compound with a structure similar to PVC. We intend to increase the interaction between DOP and PVC to suppress migration, rather than increasing molecular size (or molecular weight).

## Results

### Synthesis of the DOP-like plasticizer

**Figure 1:**
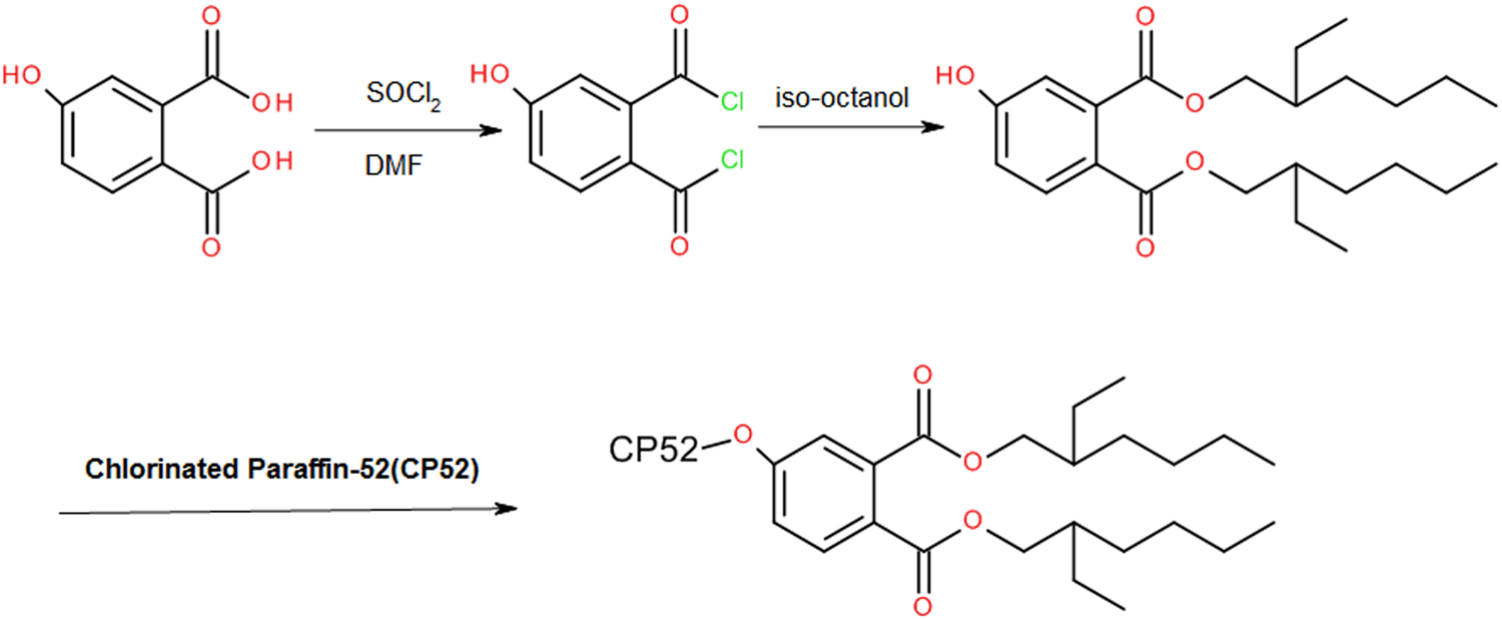
Synthetic Route of the DOP-like Plasticizer.

**Figure 2:**
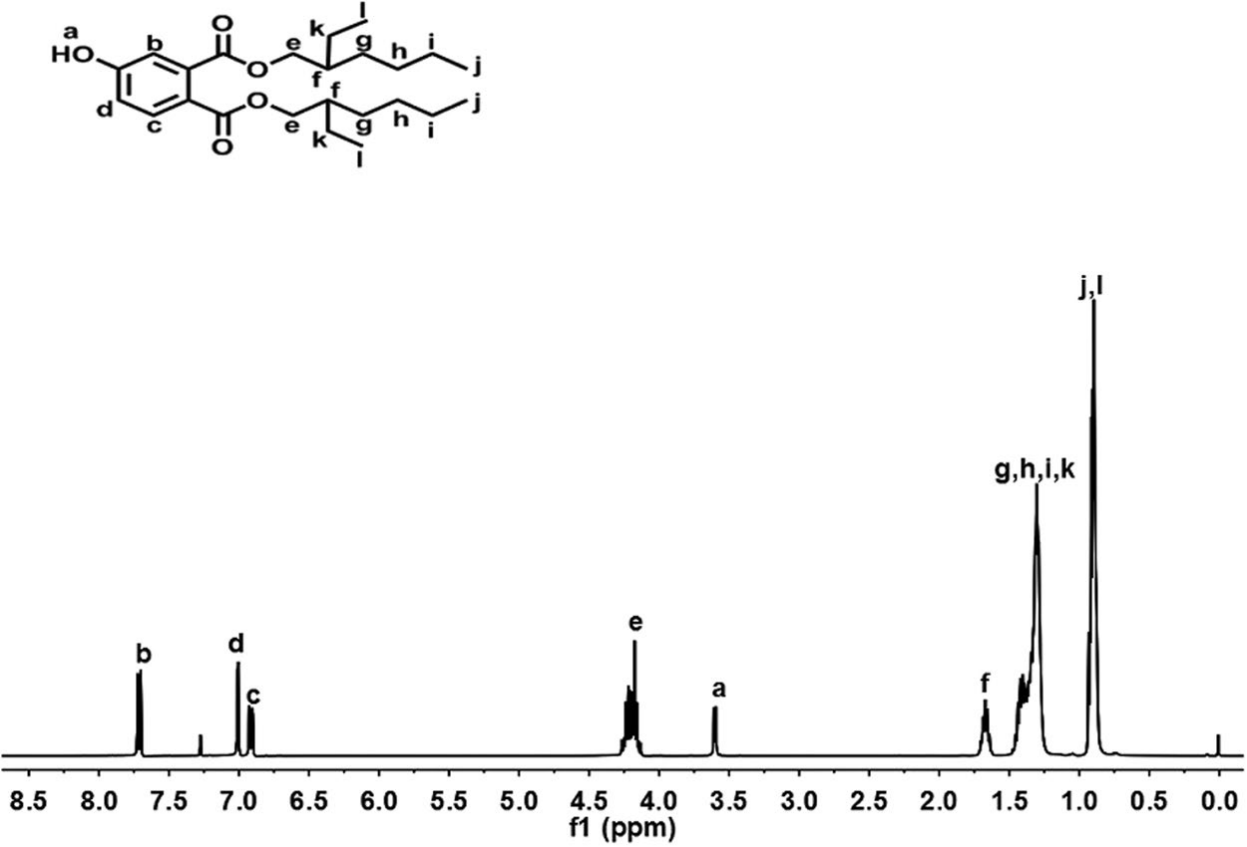
^1^H NMR spectrum of DOP-OH.

As shown in Fig. 1, a commercially available 4-hydroxyphthalic acid was converted into acyl chloride with thionyl chloride, which was then alcoholised with iso-octanol with N,N-dimethyl formamide (DMF) as an acid scavenger. Unreacted 4-hydroxyphthalic acid and iso-octanol was sequentially removed with sodium bicarbonate solution and ethylene glycol. Purification was achieved through column chromatography and the yield was 93%. The ^1^H-NMR spectrum of DOP-OH is shown in Fig. 2.

**Figure 3:**
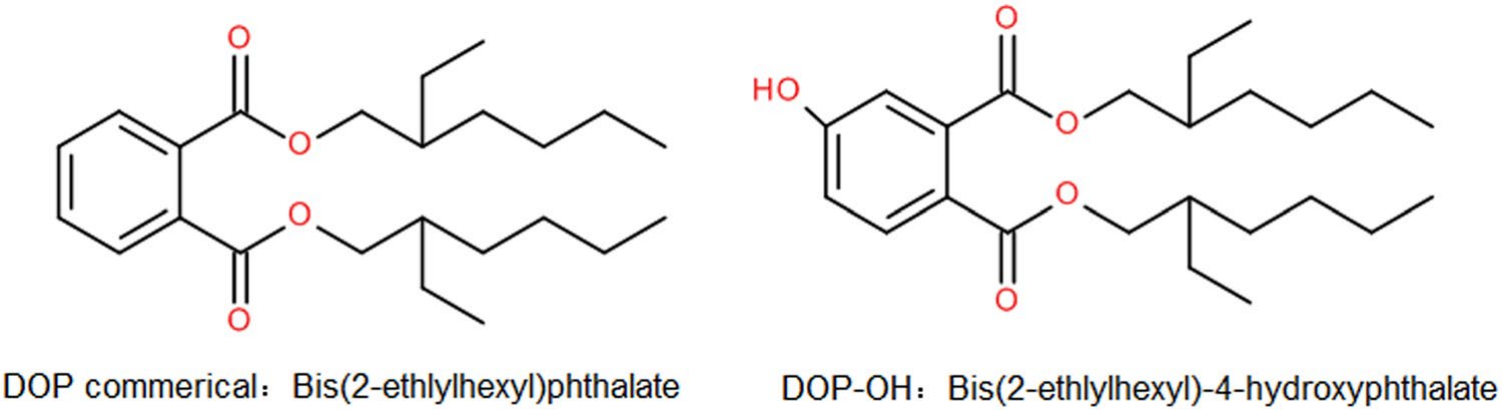
Chemical structures of DOP and DOP-OH.

**Figure 4:**
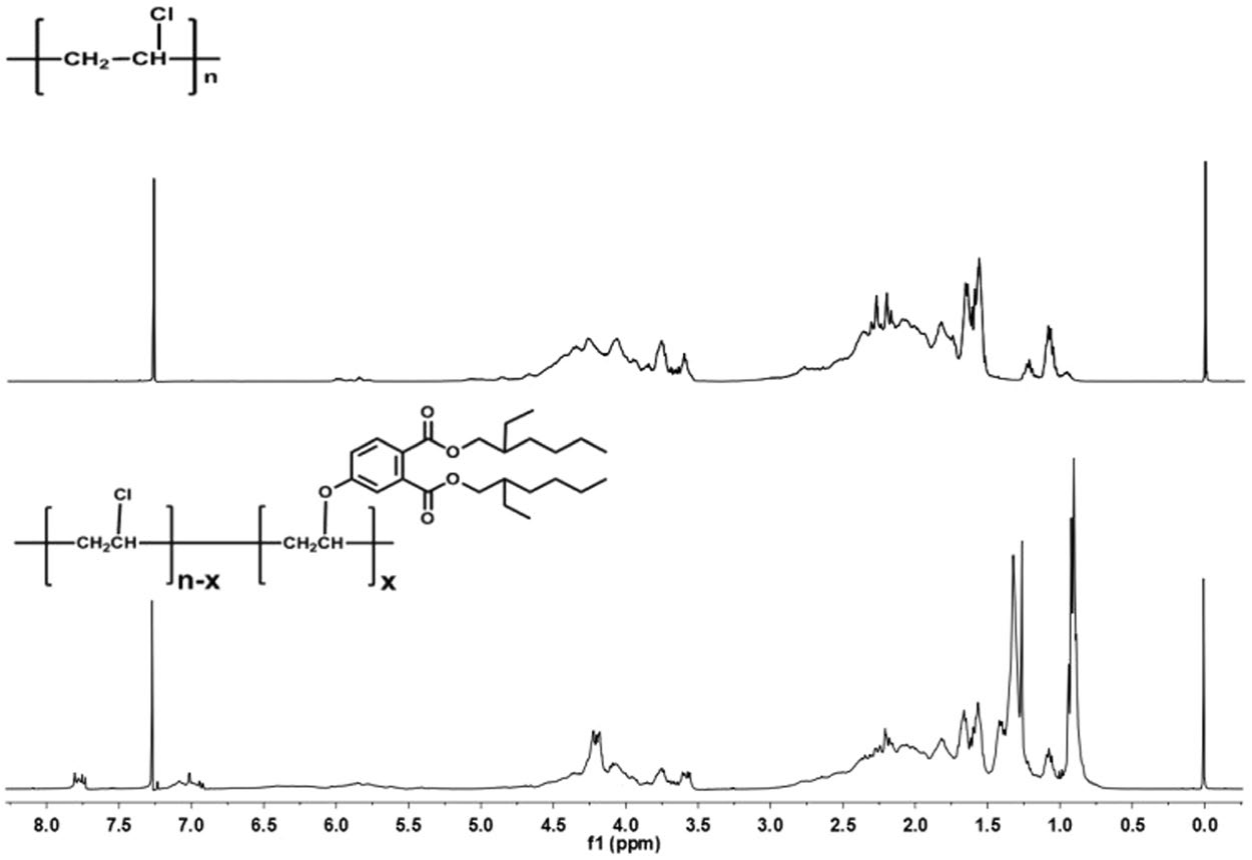
^1^H NMR spectra of CP-52 and DOP-O-CP52.

As shown in Fig. 3, DOP-OH has a similar structure to DOP, and therefore they may have similar plasticization properties. Chlorinated paraffin with chlorination levels of 52% (CP-52) was selected as an alkyl chloride because its chlorine content was similar to PVC. The purified, final DOP-like plasticizer from di(2-ethylhexyl)4-hydrophthalate and chlorinated paraffin (DOP-O-CP52) had a yield of 20.1%. As shown in Fig. 4, the proton peak of aromatic (7.0 to 8.0 ppm) and aliphatic (0.5 to 1.8 ppm) groups should be attributed to DOP-OH attached to CP-52 in DOP-O-CP52. Peaks near 4.3 ppm were from DOP-OH.

### Glass Transition Temperature

**Figure 5:**
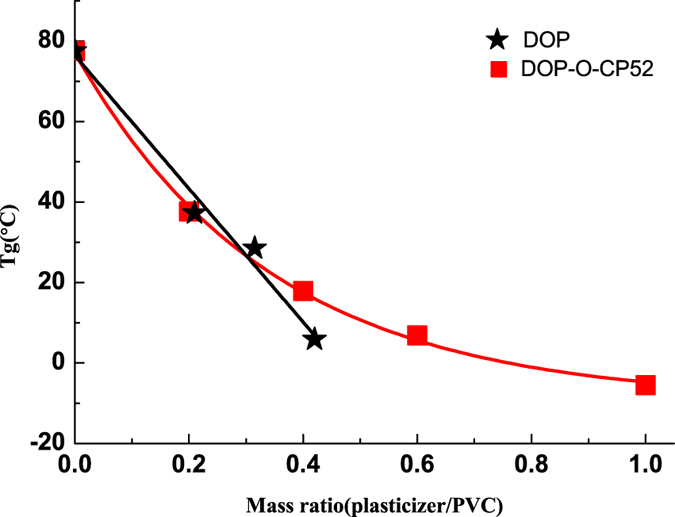
Variation of glass temperature with content of DOP (black stars) and DOP-O-CP52 (red blocks).

As shown in Fig. 5, glass temperatures (Tgs) of DOP-plasticized and DOP-O-CP52-plasticized PVC were studied with respect to the ratio of plasticizers to PVC. Both Tgs were gradually reduced with the increased addition of plasticizer. DOP-O-CP52 has almost the same plasticizing efficiency as DOP within the ratio range (from 0 to 0.3) of plasticizer to PVC, which indicated the novel DOP-like plasticizer could have the same performance as DOP in many applications. Further increases in the ratio of plasticizer over PVC led to decreased plasticizing efficiency of DOP-O-CP52 compared to DOP. However, the system with 1:1 plasticizer over PVC had a Tg below 0 °C, indicating that the system was completely flexible at room temperature and could be used for soft (flexible) PVC products.

### Migration

**Figure 6:**
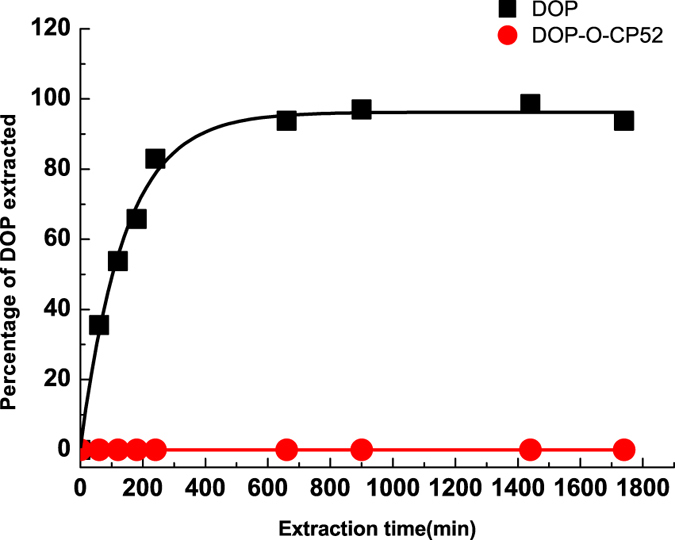
Extraction of plasticized PVC strips with heptane at room temperature (PVC/DOP (black blocks) and PVC/DOP-O-CP52 (solid red circles)).

To observe the migration of plasticizers, the migration behaviour of the new DOP-like plasticizer in PVC was tested through extraction experiments with n-heptane at room temperature^35^. As a comparison, migration of DOP was also measured under the same conditions. A small piece of plasticized PVC was placed in a bottle with n-heptane as extraction solvent and a small amount of 1, 6-hexamethylene diisocyanate as the internal reference. The solution was sampled over time. The amount of plasticizer in the solution was determined with IR spectroscopy based on the calibration curve. As shown in Fig. 6, the DOP-plasticized PVC lost almost all DOP within 4 h, but DOP-O-CP52 did not lose any DOP, even after a 30 h extraction.

## Discussion

High plasticizing efficiency of DOP for PVC is attributed to DOP’s good compatibility with PVC and its high rate of diffusion in PVC, which result from the interaction between DOP and PVC molecules. For plasticization, intermolecular forces between plasticizer molecules must be as strong as those between the plasticizer and the polymer to be plasticized^36, 37^. The stronger interaction between plasticizer molecule and PVC molecule, the greater the compatibility If intermolecular forces between the plasticizer molecules themselves is stronger than between plasticizer and polymer, the plasticizer will be less compatible with polymer, possibly losing plasticization. Although it has limited compatibility with PVC, chlorinated paraffin as a secondary plasticizer may have a stronger interaction with PVC than other secondary plasticizers because it has a chemical structure similar to PVC. In this study, DOP and chlorinated paraffin were chemically combined into a new compound, DOP-O-CP52. Enlarged molecular size will reduce its diffusion rate, resulting in the decreased plasticizing efficiency of DOP in the compound. However, besides DOP, the chlorinated paraffin parts of the new compound will also interact with PVC to mask more sites of attachment between PVC molecules and hinder forces holding PVC chains together, partially compensating the decreased plasticizing efficiency. This may why DOP-O-CP52 has such amazing plasticizing efficiency. With higher DOP-O-CP52 levels in PVC, CP52 in DOP-O-CP52 will form microclusters owing to their lower compatibility with PVC, to retard the movement of DOP-O-CP52 in PVC, resulting in a decline in plasticizing efficiency.

Figure 6 shown the extraction of plasticized PVCs. Flexible samples with 0.32:1 plasticizer over PVC were selected to ensure a high migration rate of plasticizers. These results indicated that the new DOP-like plasticizer in PVC was very stable. Compared to DOP, the larger molecular size of DOP-O-CP52 could obstruct its migration. More importantly, there was a stronger dipole-dipole interaction between PVC and DOP-O-CP52. In addition to DOP, there is also CP52 in DOP-O-CP52 which can interact with PVC molecules. Therefore, polar parts in DOP-O-CP52 were much larger than in DOP, and CP52 appears to play a key role. Combining CP52 and DOP into one molecule undoubtedly increases DOP’s interaction with PVC, resulting in DOP’s high permanence in PVC. However, stronger interactions between plasticizer and PVC will reduce its rate of diffusion in PVC, leading to lower plasticizing efficiency. A compromise is achieved using DOP-O-CP52, yielding PVC with no migration and high plasticization. CP52 appears to be a better choice for DOP. The interaction between CP52 and PVC is appropriately strong to maintain both the plasticization and nonmigration of DOP-O-CP52. Chlorinated Paraffins (CPs) are a family of complex mixtures. The chlorination degree and structure of CPs can be widely varied, and thus the interaction between CPs and PVC can be regulated. It is speculated that covalently combining phthalates and CP with appropriate chlorination degree and structure can yield a large number of plasticizers with high plasticizing efficiency and the desired nonmigrating performance of PVC.

Overall, a compound combining di(2-ethylhexyl)phthalate and chlorinated paraffin-52 was prepared. This new compound ensured the plasticity of PVC with as high plasticizing efficiency as DOP, without DOP leaking or migration. This approach covalently attaches phthalate to chlorinated paraffin, a low molecular weight compound with a structure similar to PVC. Both plasticization and nonimmigration were maintained. Based on phthalates and chlorinated paraffins with a broad range of chlorination degrees and structures, the approach is highly versatile for producing desired PVC with a permanent plasticizer effect and no leaching.

## Methods

### Materials

PVC was from Sumitomo Chemical (Japan), and 4-hydroxyphthalic acid with 98% purity % was from Wuxi Discovery Medical Technology. Thionyl chloride (SOCl_2_) was analytical grade from Tianjin Damao Chemical Reagent Factory. Iso-octanol was analytical from Tianjin Fuchen Chemical Reagents Factory. Chlorinated paraffin-52 was an experimental reagent purchased from Chengdu Kelon Chemical Reagent Factory. Sodium bicarbonate, ethylene glycol and DMF were analytical grade from Sinopharm Chemical Reagent Co., Ltd.

### Preparation of the Plasticizer

The 4-Hydroxyphthalic acid (2.2750 g, 0.0125 mol) was weighed into a three-necked flask (100 mL), and DMF (0.3 g) was added. SOCl_2_ (29.750 g, 0.250 mol, 20 equiv) was dropped with ice cooling through a constant-pressure dropping funnel. The system was heated to 60 °C for an hour. The excess SOCl2 was distilled away under reduced pressure, and iso-octanol (3.9000 g, 0.0300 mol, 2.4 equiv) was added. After the reaction was heated at 65 °C for 2 hours, the reaction solution was washed successively with distilled water, saturated sodium bicarbonate, distilled water, ethylene glycol and distilled water. After the solution was dried over anhydrous magnesium sulphate, a pale yellow liquid was obtained. It was purified by column chromatography on silica gel with petroleum ether/ethyl acetate (v/v = 5/1.5) as the eluent. The pure product (4.720 g) was obtained with a yield of 93.0%. Rf = 0.52 (petroleum ether/ethyl acetate, 5:1.5). ^1^H NMR (400 MHz, CDCl3) δ: 7.68 (d, 1H, J = 8.4 Hz, Ar-H), 7.00 (d, 1H, J = 2.0 Hz, Ar-H), 6.90 (dd, 1H, J = 8.4 and 2.4 Hz, Ar-H), 4.09–4.25 (m, 4H, 2 × COOCH2), 3.58 (s, 1H, -OH), 1.59–1.72 (m, 2H, 2 × (CH2)2CH-CH2), 1.26–1.44 (m, 16H, 8 × CH2), 0.86–0.95 (m, 12H, 4 × CH3).

CP-52 (0.6030 g) and the prepared di(2-ethylhexyl)-4-hydrophthalate hydroxyl compounds (3.2480 g, 8 mmol) were dissolved in 50 ml of cyclohexanone. After potassium carbonate was added, the reaction was performed at 65 °C in an N2 atmosphere for 24 hours. The reaction solution was washed successively with distilled water, ethylene glycol, N,N-dimethylformamide (DMF) and distilled water. After the solution was dried over anhydrous magnesium sulphate, a yellow liquid was obtained. The crude product was purified by column chromatography on silica gel with petroleum ether/ethyl acetate (v/v = 5/1.5) as the eluent. The pure product was obtained (0.653 g, 20.1%). Rf = 0.72 (Petroleum ether/ethyl acetate, 5:1.5).

### Preparation of Plasticized PVC Specimens

The plasticized PVC specimens with different amounts of plasticizer (DOP or DOP-O-CP52) were prepared. PVC powder and plasticizer were dissolved in THF, and THF was evaporated to obtain a film, which was further dried under low vacuum. For extraction experiments, 0.50 g of PVC powder and 0.16 g of plasticizer (DOP or DOP-O-CP52) were dissolved in 30 ml of THF, and THF was evaporated. After drying under vacuum, the film was cut into 15 mm × 15 mm pieces.

### Extraction Experiment

0.206 g of a plasticized PVC specimen was accurately weighed and transferred into a 10 ml volumetric flask, half-filled with n-heptane. The internal standard 1,6-hexamethylene diisocyanate(0.020 g) was added. Volume was made up to the mark with n-heptane. The flask was shaken from time to time for a predetermined time period. Two to four drops of the solution were sandwiched between two KBr windows and fixed on a holder for IR measurement. The amount of DOP extracted was determined using a calibration curve.

### Characterization

The ^1^H NMR spectrum data of the bis(2-ethylhexyl)-4-hydroxy phthalate(DOP-OH), the ester of di(2-ethylhexyl)-4-hydrophthalate and chlorinated paraffin were recorded on an AVANCE III 400 MHz spectrometer with chloroform-D as the solvent. The infrared spectrum to determine the extraction DOP was recorded on the Fourier Transform Infrared Spectrometer 8700. Tg was measured on a Perkin-Elmer differential scanning calorimeter DSC-8000. The sample (approximately 5 mg) was scanned from room temperature (25 °C) to 150 °C at 10 °C/min in a nitrogen atmosphere and quenched to −70 °C at a cooling rate of 150 °C/min, then maintained at −70 °C for 5 min. The Tg value was reported from the second run, and the midpoint of the corresponding DSC curve was obtained.

## Electronic supplementary material

Supporting Information
