# Supplementary material for: A Strategy for Nonmigrating Highly Plasticized PVC
Source: Sci Rep. 2017 Aug 24;7:9277. doi: 10.1038/s41598-017-10159-7 (PMC5570904; doi:10.1038/s41598-017-10159-7)
Supplement: Supplementary file 1 — Supporting Information [file 41598_2017_10159_MOESM1_ESM.pdf]

Supporting Information

A Strategy for Nonmigrating Highly Plasticized PVC

Jun Yuan， Bin Cheng*

Key Laboratory of Beijing City on Preparation and Processing of Novel Polymer Materials, Beijing University of Chemical Technology, Beijing100029, China

***Corresponding author:** chengb@mail.buct.edu.cn; Tel. & Fax: +86 10 64 43 47 25

Contents

1. DSC diagrams of PVCs plasticized by DOP

2. DSC diagrams of PVCs plasticized by DOP-O-CP52

3. Infrared spectra of DOP and 1,6-hexamethylene diisocyanate in Heptane solution.

4. The calibration curve of DOP dissolved in heptane

5. Infrared spectra of extraction liquid from DOP plasticized PVC

6. Infrared spectra of extraction liquid from DOP-O-CP52 plasticized PVC

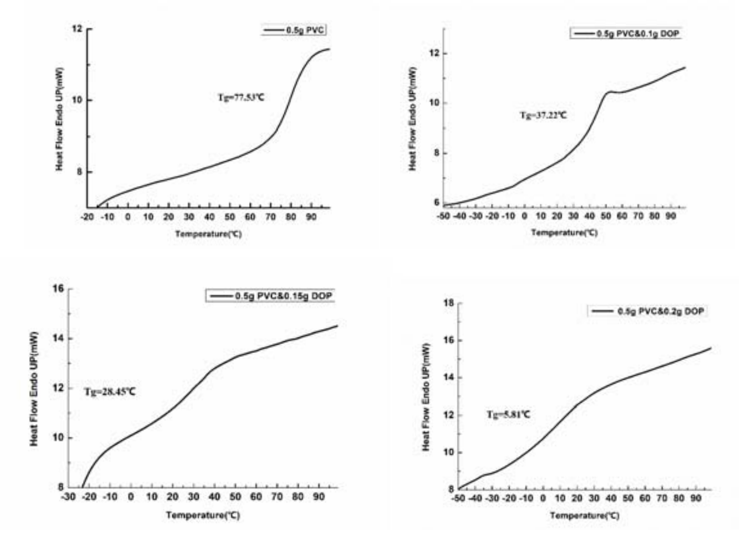

Figure1. The glass transition temperatures and compositions of PVCs plasticized by DOP

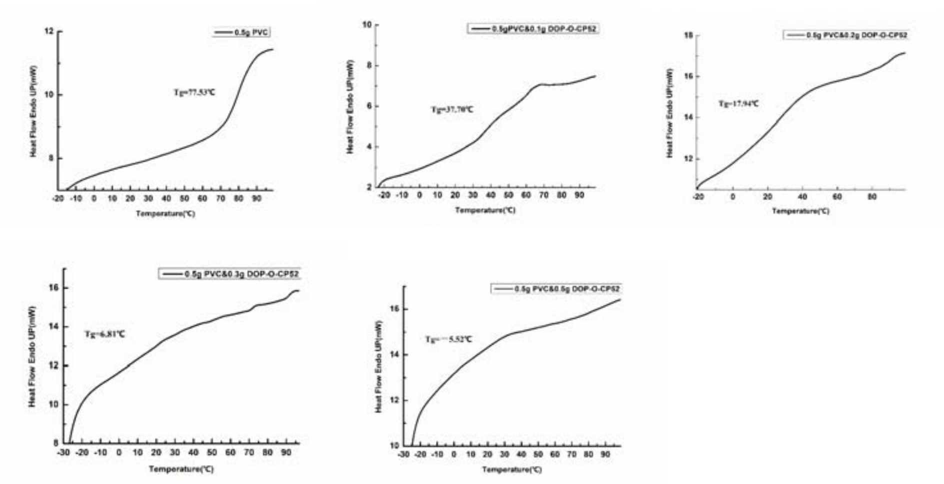

Figure2. DSC diagrams of PVCs plasticized by DOP-O-CP52.

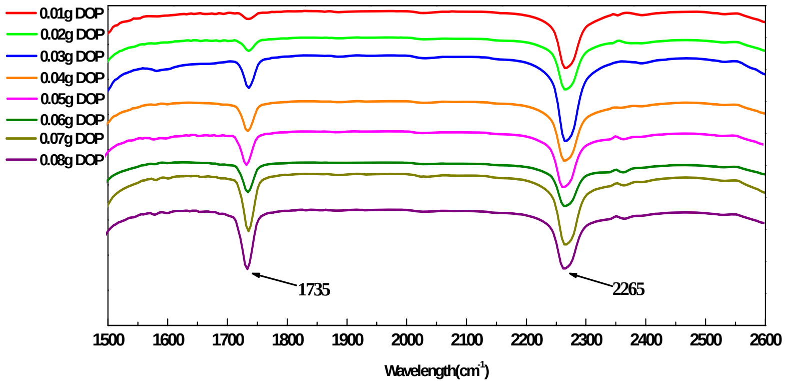

Figure3. The infrared spectra of DOP and 1,6-hexamethylene diisocyanate(0.02g) in 10ml n-heptane solution

Peaks at 1735cm $^{-1}$ and 2265cm $^{-1}$ are attributed, respectively, to the C=O of DOP and the N=C=O of 1,6-hexamethylene diisocyanate

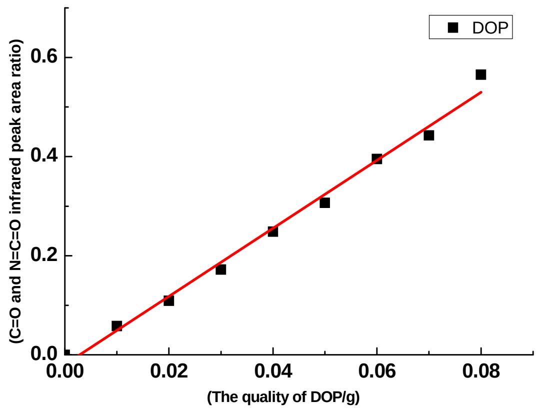

Figure4. The calibration curve of the DOP in 10ml n-heptane solution

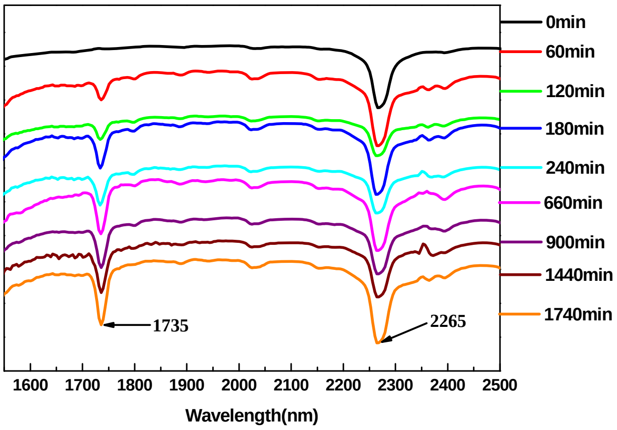

Figure5. Infrared spectra of extraction liquid from DOP plasticized PVC at different extraction time(sample composed of DOP (0.16g) and PVC (0.5g))

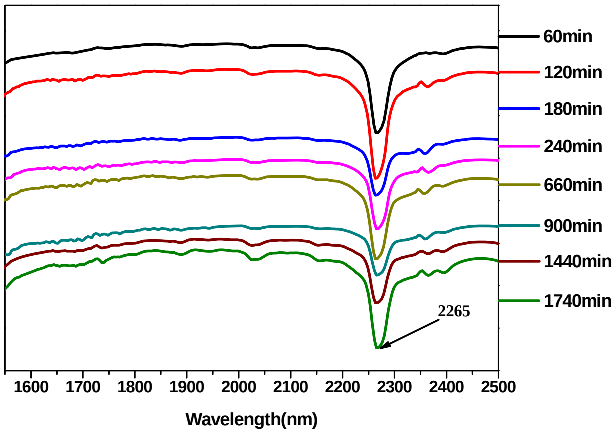

Figure6. Infrared spectra of extraction liquid from DOP-O-CP52 plasticized PVC at different extraction time(sample composed of DOP-O-CP52 (0.16g) and PVC (0.5g))
